# Ar-turmerone inhibits the proliferation and mobility of glioma by downregulating cathepsin B

**DOI:** 10.18632/aging.204940

**Published:** 2023-09-26

**Authors:** Wenpeng Cao, Xiaozong Chen, Chaolun Xiao, Dengxiao Lin, Yumei Li, Shipeng Luo, Zhirui Zeng, Baofei Sun, Shan Lei

**Affiliations:** 1 Department of Anatomy, Key Laboratory of Human Brain Bank for Functions and Diseases of Department of Education of Guizhou Province, Guizhou Medical University, Guiyang 550009, Guizhou, China; 2 Department of Neurosurgery, The Jinyang Hospital Affiliated to Guizhou Medical University, Guiyang 550009, Guizhou, China; 3 Department of Physiology, School of Basic Medicine, Guizhou Medical University, Guiyang 550009, Guizhou, China

**Keywords:** ar-turmerone, glioma, cathepsin B, proliferation, mobility, cell cycle

## Abstract

Ar-turmerone, a compound isolated from turmeric seeds, has exhibited anti-malignant, anti-aging and anti-inflammatory properties. Here, we assessed the effects of ar-turmerone on glioma cells. U251, U87 and LN229 glioma cell lines were treated with different concentrations of ar-turmerone (0, 50, 100 and 200 μM), and their viability and mobility were evaluated using Cell Counting Kit 8, colony formation, wound healing and Transwell assays. The effects of ar-turmerone on U251 glioma cell proliferation were also assessed using a subcutaneous implantation tumor model. High-throughput sequencing, bioinformatic analyses and quantitative real-time polymerase chain reactions were used to identify the key signaling pathways and targets of ar-turmerone. Ar-turmerone reduced the proliferation rate and mobility of glioma cells *in vitro* and arrested cell division at G1/S phase. Cathepsin B was identified as a key target of ar-turmerone in glioma cells. Ar-turmerone treatment reduced cathepsin B expression and inhibited the cleavage of its target protein P27 in glioma cells. On the other hand, cathepsin B overexpression reversed the inhibitory effects of ar-turmerone on glioma cell proliferation, mobility progression *in vitro* and *in vivo*. In conclusion, ar-turmerone suppressed cathepsin B expression and P27 cleavage, thereby inhibiting the proliferation and mobility of glioma cells.

## INTRODUCTION

Glioma is the most prevalent type of primary brain tumor [1], and exhibits malignant characteristics such as high aggressiveness, abnormal metabolism and angiogenesis [2]. The median survival time of glioblastoma patients is approximately fifteen months, and the recurrence rate is high [3]. The main treatment method for glioma is surgical resection, and postoperative adjuvant radiotherapy combined with chemotherapy is also an important approach [4]. Although temozolomide is an effective first-line chemotherapeutic drug, drug resistance limits its efficacy in glioma patients [5]. Therefore, it is clinically significant to develop new drugs to treat glioma.

Medicinal plants are a major source of potential drugs, and numerous plant extracts have been reported to exert anti-proliferative or anti-tumor effects [6–8]. Many drugs currently used as the gold standard in cancer treatment are of natural origin or derived from natural compounds [9, 10]. Curcuma longa L. is a plant in the Zingiberaceae family that has anti-tumor, anti-inflammatory and antioxidant effects [11, 12]. The primary active components of this plant are curcumin compounds and the volatile oil of turmeric, which mainly contains ar-turmerone (30.3%), α-curcumone (26.5%) and β-curcumone (19.1%) [13]. Ar-turmerone has been shown to suppress tumor, bacterial and fungal growth, platelet aggregation, oxidative stress, senile dementia and metabolic syndrome [14, 15]; however, its effects on glioma have not yet been reported.

Cathepsin B (CTSB) is a member of the papain-like protease family located in lysosomes [16]. It is encoded by the *CTSB* gene of human chromosome NC_000008.11, which is transcribed and translated into a CTSB proenzyme containing a signal peptide [17]. The CTSB proenzyme is cleaved by a signal peptidase in the endoplasmic reticulum to form an inactive precursor of 44 kDa. This precursor is acidified in the Golgi apparatus to generate the final active form containing two subunits (44 kDa and 5 kDa), which are then released [18]. Zhang et al. showed that knocking out *CTSB* improved the radiosensitivity of glioma cells by inducing cell cycle arrest and reducing the efficiency of homologous recombination [19]. Ho et al. found that suppressing *CTSB* using miR-140 inhibited mesenchymal transformation and enhanced the cytotoxicity of temozolomide in glioma cells [17]. These and other studies have suggested that inhibiting *CTSB* expression may be an effective strategy for cancer therapy [20, 21]. In this study, we assessed the effects of ar-turmerone on glioma and determined the underlying mechanisms, in order to provide some experimental basis for the development of new anti-glioma drugs.

## RESULTS

### Ar-turmerone inhibited glioma cell proliferation and mobility

**Figure 1:**
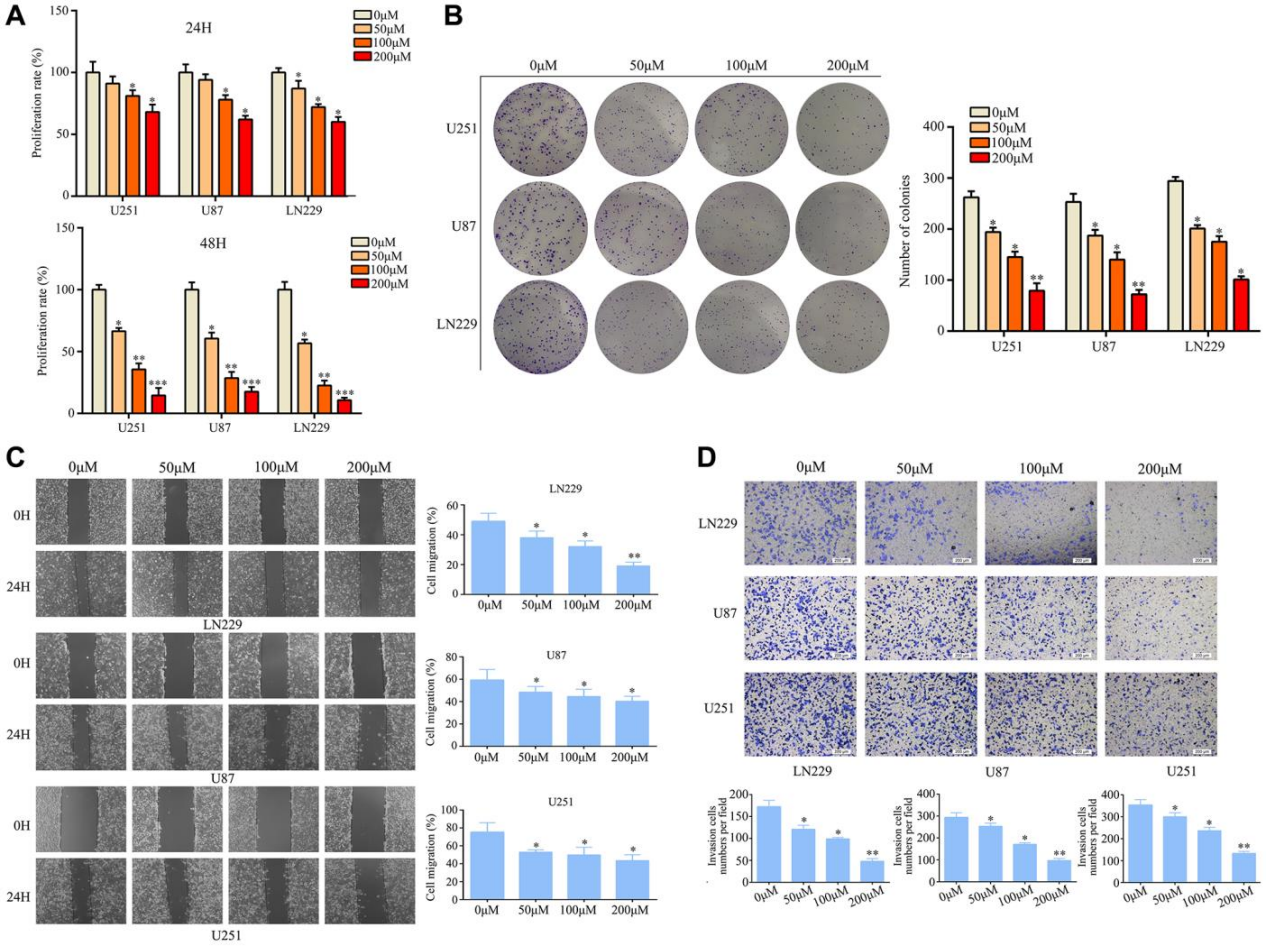
**Ar-turmerone inhibited glioma cell proliferation and mobility *in vitro*.** (**A**) U251, U87 and LN229 cells were treated with different concentrations of ar-turmerone (0, 50, 100 and 200 μM), and a CCK−8 assay was used to detect the proliferation rate of each group. (**B**) A colony formation assay was used to detect the colony formation of glioma cells treated with different concentrations of ar-turmerone (0, 50, 100 and 200 μM). (**C**) A wound healing assay was used to detect the migration of glioma cells treated with different concentrations of ar-turmerone (0, 50, 100 and 200 μM). (**D**) A Transwell assay was used to detect the invasion of glioma cells treated with different concentrations of ar-turmerone (0, 50, 100 and 200 μM). ^*^P < 0.05; ^**^P < 0.01; ^***^P < 0.001.

Various concentrations of ar-turmerone were applied to U251, U87, and LN229 cells at different time points to determine their effects on glioma cell activity. A Cell Counting Kit 8 (CCK-8) assay revealed that ar-turmerone inhibited the growth of glioma cells (Figure 1A), with significant effects at concentrations of 50, 100 and 200 μM. Colony formation assays indicated that glioma cells treated with ar-turmerone formed fewer colonies than control glioma cells after 10 days (Figure 1B). Glioma cells exhibited a reduced ability to migrate (Figure 1C) and invade (Figure 1D) after treatment with ar-turmerone. These results indicated that ar-turmerone inhibited the viability and mobility of glioma cells.

### Ar-turmerone suppressed the proliferation of glioma cells *in vivo*

**Figure 2:**
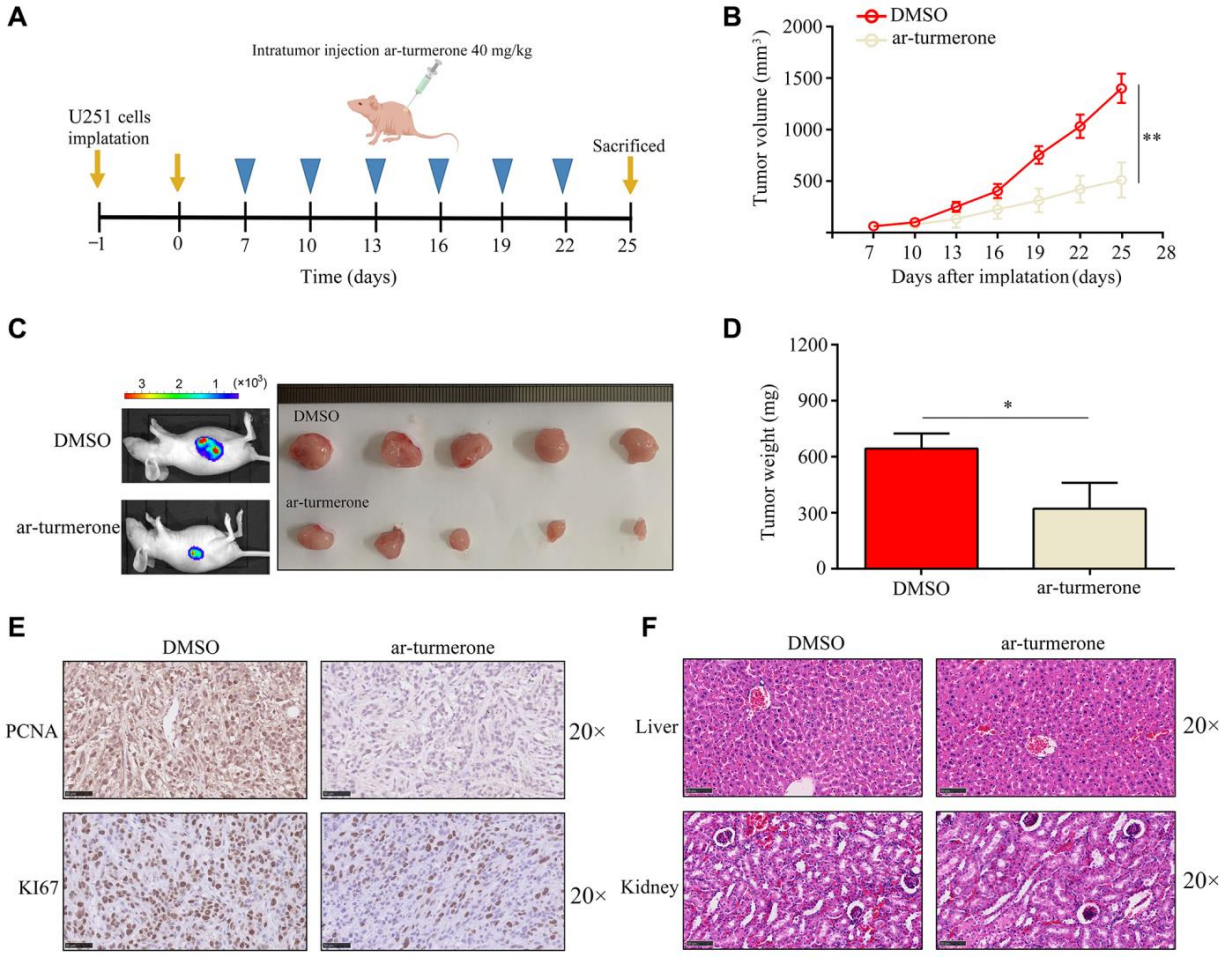
**Ar-turmerone repressed the proliferative rate of U251 cells *in vivo*.** (**A**) Model of the animal experiments. (**B**, **C**) The proliferation of tumor tissues from mice treated with DMSO or ar-turmerone. (**D**) The tumor weights of mice treated with DMSO or ar-turmerone. (**E**) PCNA and KI67 expression in tumor tissues from mice treated with DMSO or ar-turmerone. (**F**) Hematoxylin and eosin staining was performed to detect liver and kidney injuries in mice treated with DMSO or ar-turmerone. ^*^P < 0.05; ^**^P < 0.01.

Next, we assessed ar-turmerone treatment *in vivo*. BALB/c nude mice were subcutaneously injected with U251 cells, and then were intraperitoneally injected with the control treatment (dimethylsulfoxide, DMSO) or ar-turmerone (40 mg/kg). Tumor growth was rapid in the control group, whereas ar-turmerone significantly inhibited tumor growth (Figure 2A–2C) and reduced the tumor weight (Figure 2D). Compared with DMSO treatment, ar-turmerone treatment also downregulated the proliferation markers KI67 and proliferating cell nuclear antigen (PCNA) in tumor tissues (Figure 2E). Hematoxylin and eosin staining revealed no liver or kidney injury in nude mice treated with ar-turmerone (Figure 2F). Thus, ar-turmerone had obvious anti-tumor effects *in vivo*, with few toxic side effects.

### Ar-turmerone induced G1/S-phase arrest in glioma cells *in vitro*

**Figure 3:**
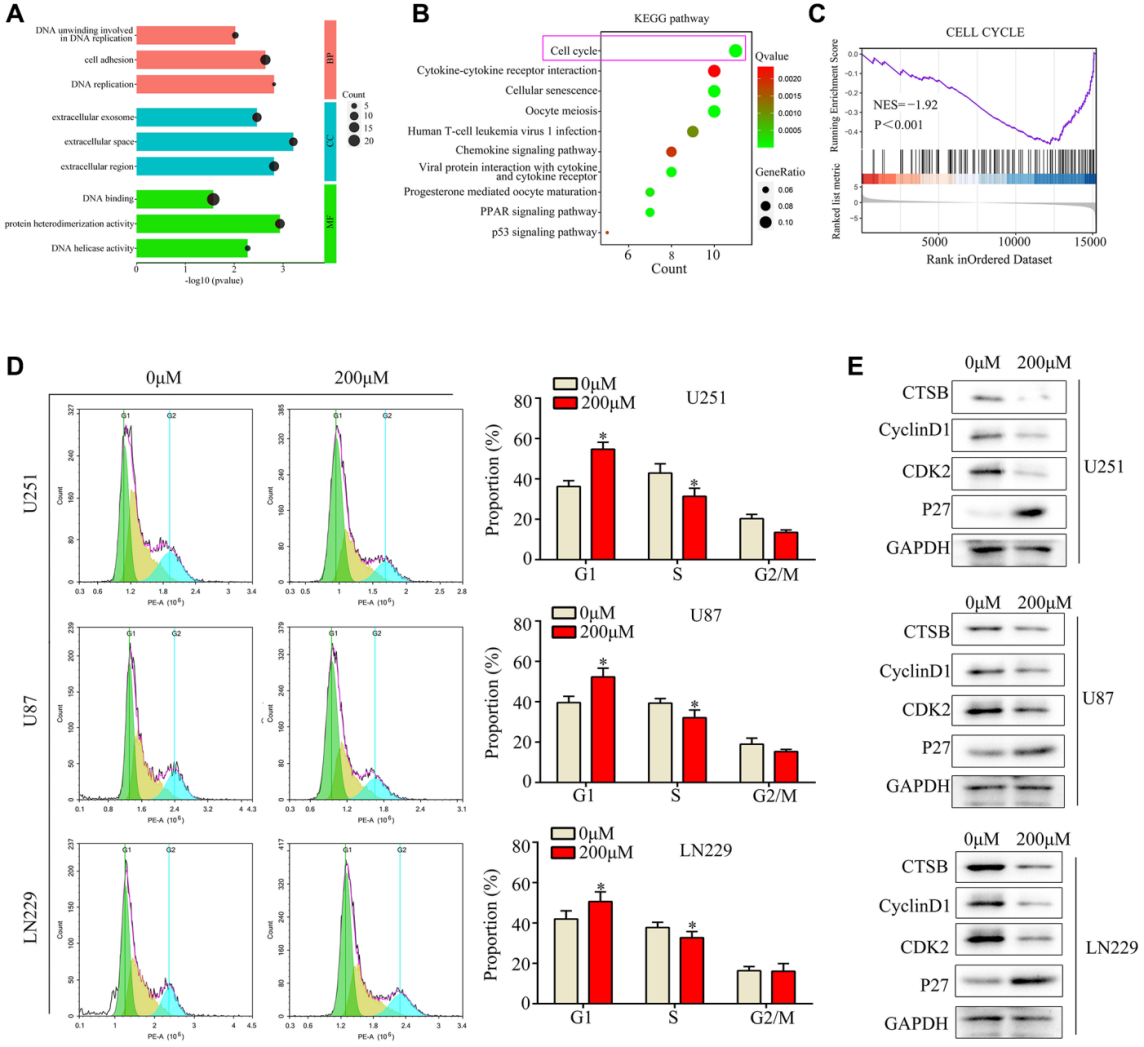
**Ar-turmerone induced glioma cell cycle arrest at G1/S phase *in vitro*.** (**A**) Gene Ontology analysis was performed to determine the enriched pathways of differentially expressed genes in ar-turmerone−treated glioma cells. (**B**) KEGG analysis was performed to determine the enriched pathways of differentially expressed genes in ar-turmerone−treated glioma cells. (**C**) Gene enrichment plots depict a series of genes enriched in the Cell cycle. (**D**) Glioma cells were treated with or the control, and flow cytometry was used to detect the cell cycle distribution in each group. (**E**) Western blotting was used to detect the expression of CDK2, P27 and CyclinD1 in glioma cells treated with ar-turmerone (200 μM) or the control. ^*^P < 0.05.

We then performed high-throughput sequencing to investigate the molecular mechanism of ar-turmerone activity in glioma cells. We identified 240 genes that were downregulated and 126 genes that were upregulated in U251 cells treated with ar-turmerone, based on a |LogFold-Change| ≥2 and an adjusted *P* < 0.05 (Supplementary Table 1). We further explored these differentially expressed genes using Gene Ontology and Kyoto Encyclopedia of Genes and Genomes (KEGG) analyses. In the Gene Ontology analysis, the Molecular Function terms associated with these genes were enriched in “DNA binding”, “protein heterodimerization activity” and “DNA helicase activity”, while the Biological Process analysis was enriched in “DNA unwinding involved in DNA replication”, “cell adhesion” and “DNA replication”, and the Cellular Component analysis was enriched in “extracellular exosome”, “extracellular space” and “extracellular region” (Figure 3A). In the KEGG analysis, the differentially expressed genes were found in the “cell cycle”, “oocyte meiosis”, “cellular senescence”, “T-cell leukemia virus 1 infection”, “viral protein interaction with cytokine and cytokine receptor”, “PPAR signaling pathway”, “progesterone-mediated oocyte maturation”, “chemokine signaling pathway” and “cytokine-cytokine receptor interaction” (Figure 3B). A Gene Set Enrichment Analysis revealed that many cell cycle genes were downregulated in the ar-turmerone treatment group (Figure 3C).

Next, we conducted a flow cytometry analysis, which demonstrated that ar-turmerone treatment significantly reduced the number of glioma cells in S phase and significantly increased the number of cells in G1 (Figure 3D). Western blot analysis revealed that ar-turmerone downregulated the G1/S-phase biomarkers Cyclin-dependent kinase 2 (CDK2), CyclinD1 and upregulated P27 in glioma cells (Figure 3E). These results indicated that ar-turmerone induced G1/S-phase arrest.

### CTSB was identified as a key target of ar-turmerone

**Figure 4:**
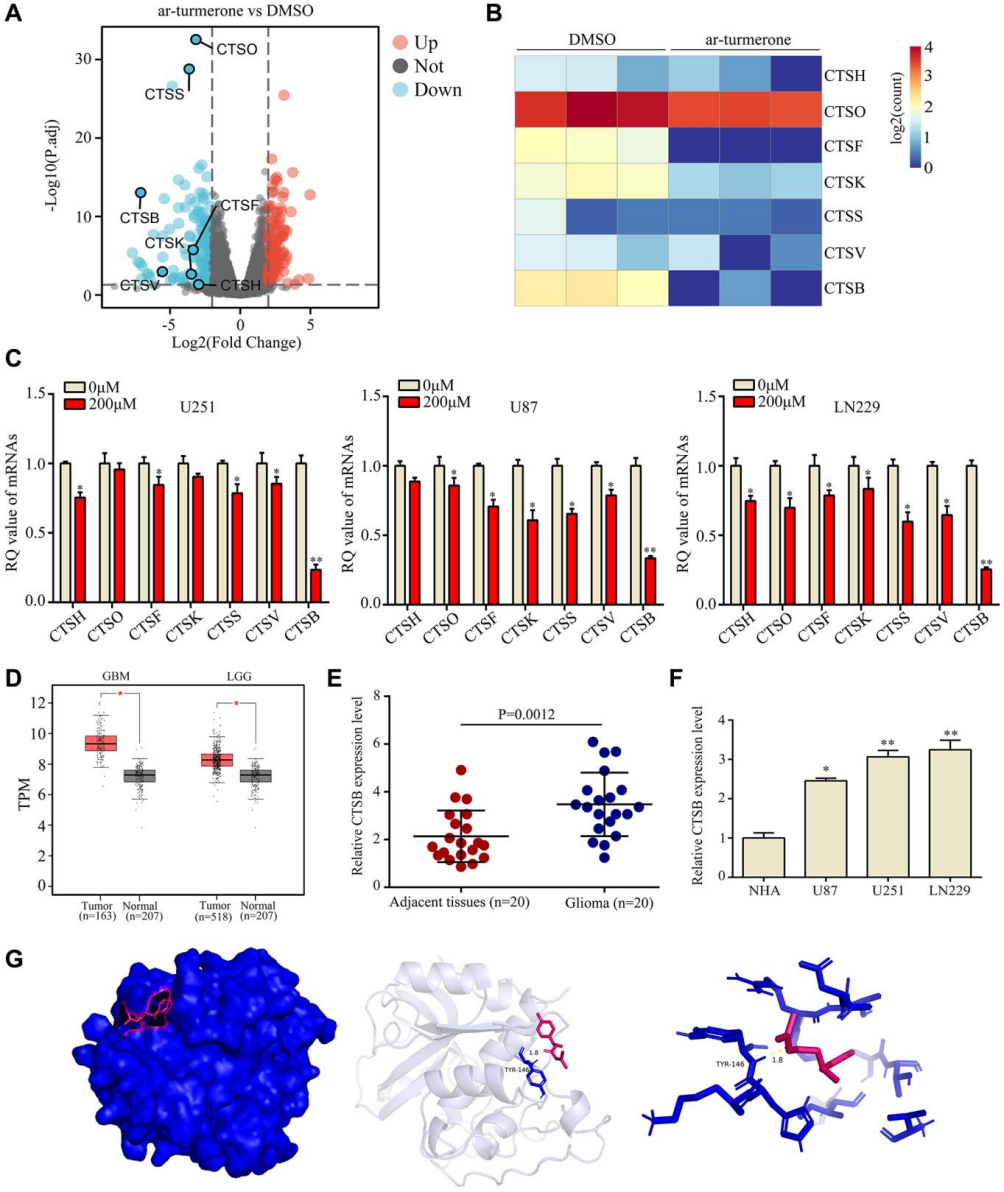
**CTSB was identified as a key target of ar-turmerone.** (**A**) Differentially expressed genes were identified in glioma cells treated with DMSO or ar-turmerone. (**B**) Heatmap showing changes in the expression of CTS family members in U251 cells. (**C**) qRT−PCR was used to detect the mRNA levels of *CTSH*, *CTSO*, *CTSF*, *CTSK*, *CTSS*, *CTSV* and *CTSB* in glioma cells treated with ar-turmerone (200 μM) or the control. (**D**) CTSB levels were significantly elevated in glioma tissues from The Cancer Genome Atlas database. (**E**) CTSB levels were significantly elevated in glioma tissues from our research cohort. (**F**) qRT−PCR was used to detect the expression of *CTSB* in normal human astrocytes, U251, U87 and LN229 cells. (**G**) The binding mode of ar-turmerone with CTSB, and the 3D illustration of the details of the interaction. Ar-turmerone is shown in blue, while the CTSB protein is shown in red. ^*^P < 0.05; ^**^P < 0.01.

Interestingly, we found seven genes in the cathepsin family among the differentially expressed genes in ar-turmerone-treated glioma cells (Figure 4A, 4B). Thus, we performed quantitative real-time PCR (qRT-PCR) analyses, which confirmed that *CTSH*, *CTSO*, *CTSF*, *CTSK*, *CTSS*, *CTSV* and *CTSB* were significantly downregulated in glioma cells treated with ar-turmerone (Figure 4C). We then conducted bioinformatic Gene Expression Profiling Interactive Analysis using data from The Cancer Genome Atlas and Genome-Tissue Expression databases, and found that CTSB expression was elevated in glioma tissues (Figure 4D). In our own research cohort, we also found that the CTSB mRNA expression was higher in glioma tissues than in adjacent tissues (Figure 4E). Additional qRT-PCR analyses revealed that CTSB levels were higher in glioma cell lines (U87, U251 and LN229) than in normal human astrocytes (NHA cells; Figure 4F).

Next, we used molecular docking technology to determine whether ar-turmerone could bind to the CTSB protein. According to the 3D drawing, ar-turmerone binds to TYR146 of CTSB in a stabilizing manner (Figure 4G). These results illustrated that CTSB is a critical target of ar-turmerone.

### Overexpressing CTSB reversed the inhibitory effects of ar-turmerone on glioma cell proliferation and mobility

**Figure 5:**
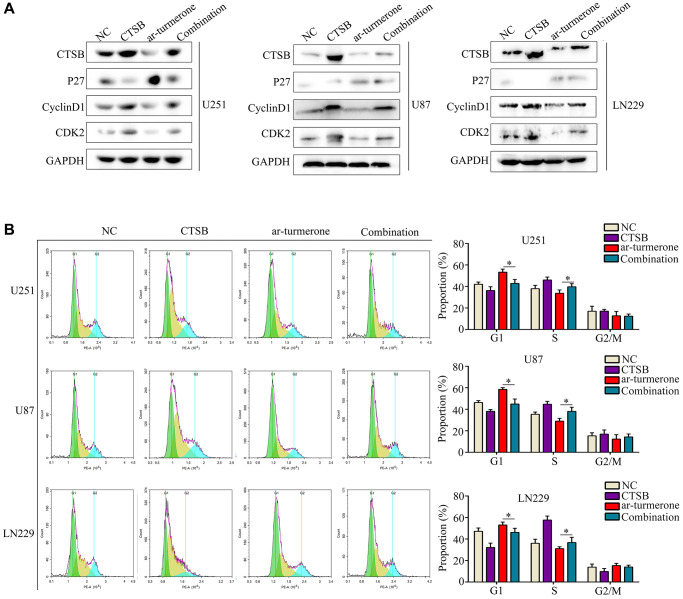
**Overexpressing CTSB prevented G1/S−phase cell cycle arrest in ar-turmerone−treated glioma cells.** Glioma cells were treated with DMSO, ar-turmerone, *CTSB* plasmids or ar-turmerone + *CTSB* plasmids, respectively. (**A**) Western blotting was used to detect the expression of CTSB, P27, CDK2 and CyclinD1 in each group of cells. (**B**) Flow cytometry was performed to detect the cell cycle distribution of glioma cells in each group. ^*^P < 0.05.

CTSB has been shown to cleave P27 and promote cell cycle arrest; thus, we assessed the expression of proteins downstream of CTSB in glioma cells after ar-turmerone treatment. Ar-turmerone treatment reduced the expression of CTSB, CDK2 and CyclinD1, but increased the expression of P27 in glioma cells. These results suggested that ar-turmerone reduces the expression of CTSB and inhibits its cleavage of P27. To confirm these findings, we overexpressed CTSB in glioma cells with and without ar-turmerone treatment. We found that CTSB overexpression partially increased CDK2 and CyclinD1 expression in ar-turmerone-treated glioma cells, while it reduced P27 expression (Figure 5A). Therefore, CTSB may be a target of ar-turmerone, such that P27 cleavage is inhibited.

**Figure 6:**
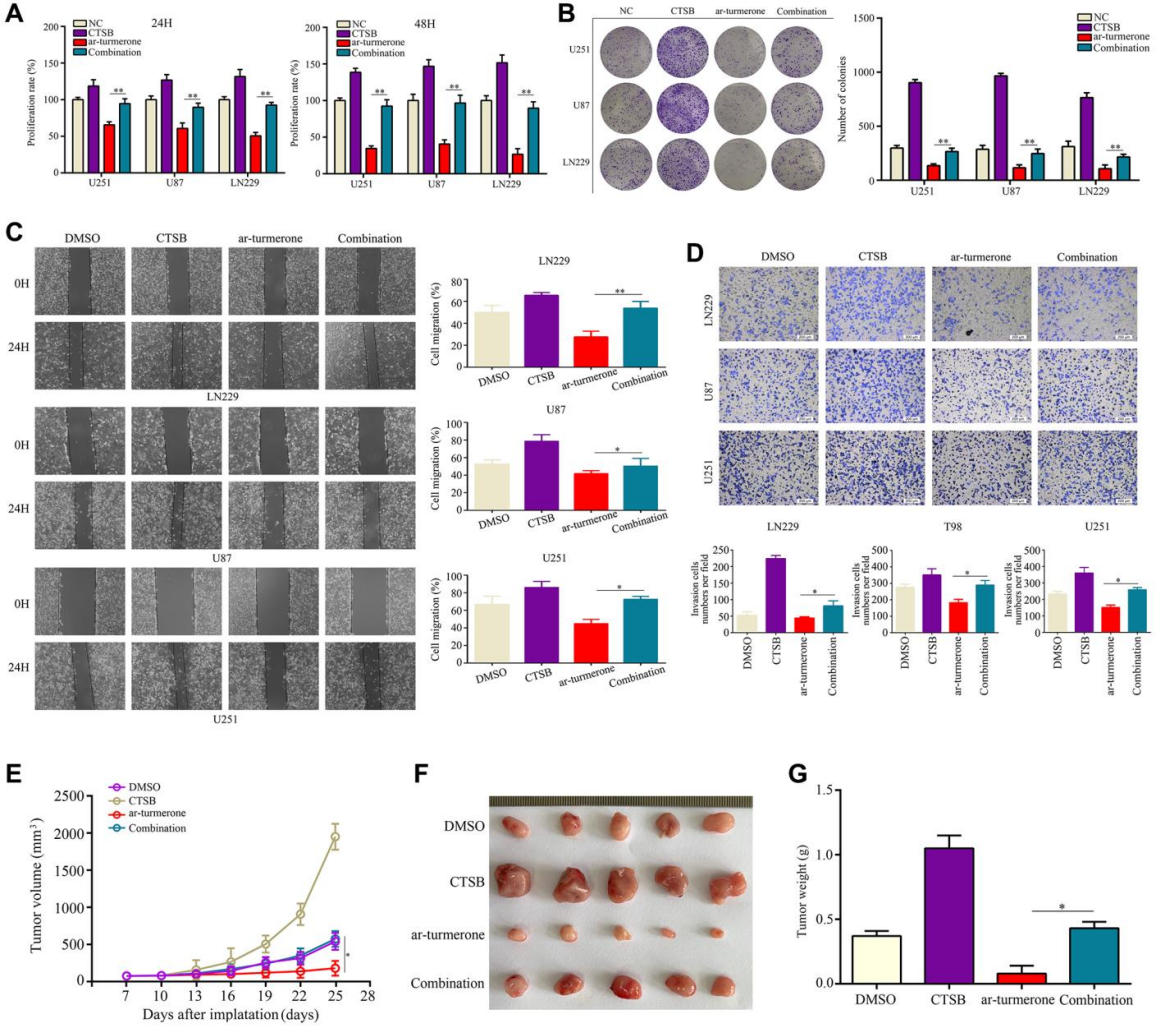
**Overexpressing CTSB reversed the inhibitory effects of ar-turmerone on glioma cell proliferation and mobility.** Glioma cells were treated with DMSO, ar-turmerone, *CTSB* plasmids or ar-turmerone + *CTSB* plasmids, respectively. (**A**) CCK−8 was used to detect the proliferative rate of glioma cells in each group. (**B**) A colony formation assay was used to detect the colony formation of glioma cells in each group. (**C**) A wound healing assay was used to detect the migration of glioma cells in each group. (**D**) A Transwell assay was used to detect the invasion of glioma cells in each group. (**E**, **F**) Subcutaneous tumorigenesis experiments were used to detect the proliferation of tumor tissues in each group. (**G**) Tumor weights of each group. ^*^P < 0.05, ^**^P < 0.01.

We then conducted a flow cytometry analysis, which demonstrated that CTSB overexpression significantly reversed the inhibitory effects of ar-turmerone on G1/S phase in glioma cells (Figure 5B). CCK-8 and colony formation assays showed that CTSB overexpression prevented ar-turmerone treatment from inhibiting glioma cell proliferation (Figure 6A) and colony formation (Figure 6B), respectively. Wound healing and Transwell assays respectively indicated that the inhibitory effects of ar-turmerone on glioma cell migration (Figure 6C) and invasion (Figure 6D) were alleviated upon CTSB overexpression. Subcutaneous tumorigenesis experiments revealed that CTSB overexpression disinhibited glioma cell tumor growth in ar-turmerone-treated mice (Figure 6E, 6F), leading to greater tumor weights (Figure 6G). These results indicated that ar-turmerone inhibits glioma cell proliferation and mobility by downregulating CTSB expression.

## DISCUSSION

Natural products are potential therapeutic options for various tumors, including glioma [22–24]. Turmeric, a traditional Chinese medicine, is rich in curcumin and turmeric volatile oil [25]. Studies have confirmed that curcumin and a variety of curcumin compounds can inhibit cell growth and migration, induce DNA damage and promote early apoptosis in glioma cells; thus, these compounds have been identified as potential anti-glioma drugs [26, 27]. Majchrzak et al. demonstrated that sodium butyrate increased the permeability of curcumin through the blood-brain barrier, thus restoring the expression of Wnt/β-catenin pathway antagonist genes and reducing the vitality of glioblastoma cells [28]. Liu et al. found that β-elemene enhanced the radiosensitivity and chemical sensitivity of glioblastoma cells by inhibiting the ataxia telangiectasia mutated signaling pathway [26]. Su et al. reported that demethoxycurcumin prevented the proliferation, migration and invasion of human glioblastoma multiforme glioma cells [29]. However, the effects of turmeric volatile oil on glioma have not yet been reported. Here, we assessed the anti-glioma activity of ar-turmerone, the main component of turmeric volatile oil. We found that ar-turmerone inhibited the proliferation, mobility and cell cycle progression of U87, U251, and LN229 glioma cells.

To determine the anti-glioma mechanism of ar-turmerone, we used RNA sequencing technology to observe the molecular landscape and biological function of glioma cells treated with ar-turmerone. We observed significant changes in 366 genes, with enrichment for cell cycle genes that were significantly downregulated. Interestingly, the downregulation of *CTSB* was the most significant in glioma cells treated with ar-turmerone.

As a cysteine proteolytic enzyme, CTSB can lyse the extracellular matrix and basement membrane and destroy the host interstitial barrier, thus facilitating the invasion of various tumors [30, 31]. In addition, CTSB maintains transfer-related protease activity through damage-related inhibitors, thereby promoting extracellular matrix degradation and angiogenesis [21]. In colorectal cancer, suppressing CTSB expression was found to downregulate Cyclin B1 and upregulate the Cyclin-CDK inhibitory protein P27kip1 in G2 phase, thereby inhibiting tumor growth [32]. In hepatocellular carcinoma cells, CTSB was shown to activate the phosphoinositide 3-kinase/Akt pathway, thereby promoting tumor invasion by increasing matrix metallopeptidase 9 expression [33, 34].

In this study, we observed that CTSB mRNA levels in glioma cells decreased following ar-turmerone treatment *in vitro*. Additionally, our *in vivo* studies demonstrated that ar-turmerone suppressed glioma cell proliferation and reduced KI67 and PCNA expression in tumor tissues. We found that CTSB expression was higher in glioma tissues than in adjacent tissues, and that CTSB overexpression significantly disinhibited the proliferation and migration of ar-turmerone-treated glioma cells. Overall, our results suggested that ar-turmerone suppresses glioma by inhibiting CTSB.

## MATERIALS AND METHODS

### Cell culture

The U251, U87 and LN229 cell lines were obtained from the American Type Culture Collection (ATCC, USA) [35]. The cells were cultured in Dulbecco’s modified Eagle’s medium (DMEM; Gibco, USA) containing 10% fetal bovine serum (FBS; Bioink, Israel) at 37°C with 5% CO_2_. Ar-turmerone powder (MCE, Wuhan, China) was mixed with DMSO to form a 10 mol·L^−1^ mother liquor, which was then filtered, sterilized and stored at −20°C. The working solution was diluted to various concentrations in the DMEM culture solution. The *CTSB* plasmids were constructed by Shanghai Kei Lei Biological Technology Co., Ltd. and were transfected into cells using Lipofectamine 2000 (Thermo Fisher Scientific, USA).

### CCK-8 assay

Glioma cells in the logarithmic growth phase were digested with trypsin to generate a cell suspension. Each well of a 96-well plate was seeded with 3 × 10^3^ cells, and six holes were punched in each group. After 48 h of treatment, the medium in each well was removed and replaced with 100 μL of serum-free DMEM mixed with 10 μL of CCK-8 solution. The cells were cultured in an incubator for 2 h, and then a microplate reader was used to measure the absorbance of each well at 450 nm.

### Colony formation assay

A six-well plate was used to cultivate a total of 800 glioma cells for 14 days at 37°C. Then, the medium was discarded and the cell colonies were fixed with 4% paraformaldehyde. The cells were washed with phosphate-buffered saline and stained with a 0.5% crystal violet solution (Thermo Fisher Scientific) for 30 min. Then, the cell colonies were counted.

### Wound healing assay

Glioma cells were seeded in six-well plates and cultured until the degree of convergence was >95%. Then, a 200-μL pipet tip was used to vertically scratch the cell monolayer to generate a wound. The floating cell mass was washed away and FBS-free medium was added. Then, the wound conditions at 0 and 24 h were recorded under an inverted optical microscope (Axiovert 200; Carl Zeiss, Germany). The migration rate of the glioma cells was determined based on the wound healing area.

### Transwell assay

FBS-free DMEM was used to resuspend glioma cells to a density of 1 × 10^6^ cells/mL, and 200 μL of this cell suspension was added to the upper chamber of a Transwell plate (Invitrogen, USA) pre-coated with Matrigel (Invitrogen). Then, 600 μL of DMEM containing 10% FBS was added to the lower chamber. The lower chamber was treated with 4% paraformaldehyde for 20 min and with 0.5% crystal violet for 15 min, followed by scrubbing. Finally, invasive cells in the lower chamber were observed using an orthotopic light microscope (Axioscope5; Carl Zeiss).

### Western blot

Total proteins were lysed with a potent precooled lysate containing 2% phenylmethanesulfonyl fluoride protease inhibitor. After centrifugation, the protein supernatants were collected and quantified using the bicinchoninic acid method. The proteins were subjected to 10% gel electrophoresis and then transferred to polyvinylidene difluoride membranes at a constant voltage of 90 V and a constant current of 300 mA for 2 h. CyclinD1 (60186-1-IG, Proteintech, China), CDK2 (10122-1-AP, Proteintech), P27 (25614-1-AP, Proteintech), CTSB (12216-1-AP, Proteintech) and GAPDH (60004-1-Ig, Proteintech) primary antibodies diluted 1:1000 were applied to the membranes for 2 h at room temperature and then overnight at 4°C. The membranes were washed three times and then treated with secondary antibodies (1:2000 dilution) for 2 h at room temperature. A high-sensitivity enhanced chemiluminescence exposure solution was added and developed using an imager. After the gray values of the bands were measured, the relative levels of the proteins were calculated using GAPDH as an internal reference.

### Subcutaneous tumorigenesis experiments

A suspension of U251 cells (2 × 10^6^ cells per 200 mL) was injected subcutaneously into the armpit of the right forelimb of each nude mouse. On the seventh day, Vernier calipers were used to determine the length and width of each subcutaneous tumor, and the tumor volume (mm^3^) was calculated as follows: (length × width^2^)/2. Mice with tumors of 40–60 mm^3^ were retained for further studies, in which they were intraperitoneally injected every three days with DMSO or ar-turmerone (40 mg/kg/day). The tumor volume was determined every three days, and used to plot the tumor growth curve. After 25 days of treatment, the mice in each group were sacrificed, their tumor tissues were removed, and their tumor weights were measured. Kidney, liver and tumor tissues were collected for further study.

### qRT-PCR

TRIzol (Thermo Fisher Scientific) was used to extract RNA from tissues and cells. The total RNA was washed with 70% ethanol and diluted with enzyme-free water. A spectrophotometer was used to determine the concentration and purity of 1 μL of the diluted RNA, and samples with purity values of 1.8–2.0 qualified for reverse transcription and PCR. A TaKaRa kit was used to reverse-transcribe 1 μg of total RNA according to the manufacturer’s instructions. The resulting cDNA was used as a template for qRT-PCR, which was conducted according to the instructions of the kit manufacturer purchased from Takara, Japan. The relative expression of each gene was calculated using the 2^−ΔΔC^^t^ method, with *GAPDH* as an internal reference. The following primers were used:

**Table:** 

*CTSB*	
Forward:	5′-GAGCTGGTCAACTATGTCAA|CA-3′,
Reverse:	5′-GCTCATGTCCACGTTGTAGAAGT-3′;
*CTSH*	
Forward:	5′-CAAGTCATGGATGTCTAAGCACC-3′,
Reverse:	5′-CATTGTTGTGGGCGTTTATCTTC-3′;
*CTSO*	
Forward:	5′-GCCTTCCGGGAAAGTCTTAATAG-3′,
Reverse:	5′-TCCAGTCAAATCTTAACGGCAAA-3′;
*CTSF*	
Forward:	5′-AGCCCAAGTCAGCCTTCAC-3′,
Reverse:	5′-CGCACCATGTTATTGACAAAGAC-3′;
*CTSK*	
Forward:	5′-ACACCCACTGGGAGCTATG-3′,
Reverse:	5′-GACAGGGGTACTTTGAGTCCA-3′;
*CTSS*	
Forward:	5′-AAACGGCTGGTTTGTGTGC-3′,
Reverse:	5′-CAGTGGTGATCCAGGGTAGG-3′;
*CTSV*	
Forward:	5′-AAACGGCTGGTTTGTGTGC-3′,
Reverse:	5′-CAGTGGTGATCCAGGGTAGG-3′;
*GAPDH*	
Forward:	5′-GGAGCGAGATCCCTCCAAAAT-3′,
Reverse:	5′-GGCTGTTGTCATACTTCTCATGG-3′.

### Flow cytometry

Glioma cells in all treatment groups were cultured in six-well plates for 48 h, digested with trypsin, centrifuged, collected and washed twice with phosphate-buffered saline. The cells were fixed with 75% ethanol overnight, and then the supernatant was centrifuged at 1000 rpm for 5 min. The cells were washed with phosphate-buffered saline to remove the residual ethanol, and then were stained with propidium iodide. The cells were incubated at 4°C in the dark for 30 min, and then were subjected to flow cytometry detection and analysis.

### RNA sequencing

TRIzol reagent was used to extract total RNA from samples. A Bioanalyzer 2100 and RNA 6000 Nano LabChip Kit (Agilent, USA) were used to determine the RNA quantity and purity, and RNA samples with RNA integrity numbers >7.0 were used to create sequencing libraries. Reverse transcription was performed using SuperScript™ II Reverse Transcriptase (Invitrogen), and the mRNA was fragmented into short pieces following purification. The cDNA inserts averaged 300 ± 50 base pairs for the final library. The cDNA was sequenced using an Illumina Novaseq™ 6000 (LC-Bio Technology, Ltd., Hangzhou, China) in accordance with the vendor’s protocol. After high-quality clean reads were obtained and batch normalization was performed, the count data were analyzed using the EdgeR package. *P* values < 0.05 and |LogFold-Change| values ≥2 were used as test criteria for differentially expressed genes [36].

### Statistical analysis

All results were analyzed using SPSS software (version 19.0). Student’s *t*-test was used to analyze the difference between two groups, while one-way analysis of variance combined with the least significant difference *t*-test was used to compare multiple groups. The significance threshold was set at *P* < 0.05.

### Availability of data and materials

The datasets used and/or analyzed during the current study are available from the corresponding author on reasonable request.

## Supplementary Materials

Supplementary Table 1
