# Supplementary material for: Ar-turmerone inhibits the proliferation and mobility of glioma by downregulating cathepsin B
Source: Aging (Albany NY). 2023 Sep 26;15(18):9377–90. doi: 10.18632/aging.204940 (PMC10564430; doi:10.18632/aging.204940)
Supplement: Supplementary Table 1 [file aging-15-204940-s001.docx]

| **Supplementary Table 1. All the differently expressed genes (DEGs) between the ar-turmerone treatment group and the DMSO treatment group are shown.** | | |
| --- | --- | --- |
| gene_name | logFC | pvalue |
| TIGD1 | -2.170618845 | 0.46713744 |
| AL139300 | -2.165906807 | 0.395110207 |
| AC080112 | -2.007999155 | 0.389950345 |
| C4orf47 | -2.368906886 | 0.27360159 |
| USH1G | -3.903051683 | 0.257341053 |
| AC105001 | -2.377637899 | 0.252610508 |
| VEZF1P1 | -2.192240154 | 0.196094292 |
| TUBA3D | -2.358143826 | 0.190045278 |
| LINC00987 | -2.098239738 | 0.149363866 |
| AL049650 | -2.172848668 | 0.146100224 |
| RBM5-AS1 | -3.208607541 | 0.134014834 |
| AC084756 | -2.352542806 | 0.131852009 |
| SNX22 | 2.150027632 | 0.131541705 |
| AC006213 | -2.996280962 | 0.123799162 |
| AC145343 | -2.448009855 | 0.118417072 |
| TNFRSF8 | -2.081647981 | 0.112682412 |
| ABCA1 | -2.388945318 | 0.103706045 |
| NTN3 | -2.395906353 | 0.079806215 |
| AP000331 | -2.623465626 | 0.072033113 |
| AC072054 | -2.096061778 | 0.070811211 |
| AC004080 | -2.000696488 | 0.067781822 |
| AC129492 | 2.053283133 | 0.067696348 |
| Z97633 | -2.457705539 | 0.06737754 |
| MIR6765 | -2.156285438 | 0.066104136 |
| RAB19 | -3.507300214 | 0.064945446 |
| CCDC116 | -2.032598379 | 0.062338821 |
| FGF1 | -2.368539918 | 0.061374582 |
| ESM1 | -2.704901861 | 0.060240499 |
| FRG1-DT | -2.194300077 | 0.060073848 |
| AC025048 | -7.376329207 | 0.059239561 |
| AC109460 | 2.236900363 | 0.058551254 |
| AC132872 | -2.245337376 | 0.05737907 |
| AL451136 | -2.55445967 | 0.052168623 |
| AL031716 | -3.138509114 | 0.047369293 |
| DCST1 | -2.524476881 | 0.043585921 |
| AL023803 | -2.918228716 | 0.043459635 |
| XYLT1 | 3.293966756 | 0.039184778 |
| C1QTNF2 | -2.218717112 | 0.038992792 |
| AC097658 | -2.103102406 | 0.03858748 |
| ATXN2-AS | -2.200820775 | 0.038121549 |
| LINC02595 | -2.051396643 | 0.037444616 |
| AP002990 | -2.212279268 | 0.037281295 |
| CXCR6 | -2.379912625 | 0.036970526 |
| FAM110D | -2.245531079 | 0.036643858 |
| AC010323 | 3.016469344 | 0.032249446 |
| AC007383 | -3.794431058 | 0.031504891 |
| ART5 | -3.16012118 | 0.030428259 |
| ITFG2-AS1 | -2.512992056 | 0.028596671 |
| AC069148 | -2.396884402 | 0.028587715 |
| ARMCX7P | -3.32594463 | 0.026436577 |
| COL6A3 | 2.097253824 | 0.025636161 |
| RASGRP2 | -3.502541123 | 0.025313843 |
| HAS2-AS1 | -2.773383062 | 0.024705143 |
| TJP3 | -2.439072345 | 0.02377102 |
| AC079781 | -2.528879779 | 0.02351109 |
| AC087721 | -8.958096925 | 0.02188901 |
| TRBV12-4 | -3.630660299 | 0.021524851 |
| AC125611 | -2.47754369 | 0.018666921 |
| MIR155HG | -2.984182169 | 0.017687301 |
| TMEM37 | -5.962011864 | 0.016008065 |
| ACTBP11 | -2.453476652 | 0.015981039 |
| AC012313 | -2.475281207 | 0.015682239 |
| SELPLG | 2.430346982 | 0.014954338 |
| AL807752 | -4.031659089 | 0.014598111 |
| CA9 | -3.103962254 | 0.013701077 |
| AC138904 | -2.460791693 | 0.013370942 |
| KRTAP2-3 | -3.709032739 | 0.012610845 |
| LSP1 | -6.176868862 | 0.012302053 |
| POU5F1B | -2.627429793 | 0.012294116 |
| AL109917 | -2.50269241 | 0.011920643 |
| FAM3B | -6.154749651 | 0.010767062 |
| CTSH | -2.960332794 | 0.01060555 |
| SYN1 | -3.131946455 | 0.010136089 |
| COL7A1 | 3.891975189 | 0.009476977 |
| TMEM269 | 2.010518201 | 0.009355446 |
| AC010655 | -2.555468783 | 0.009255842 |
| CYP4F3 | -2.809985923 | 0.008856666 |
| AC106820 | -2.375654738 | 0.007614374 |
| CCN2 | 2.042471923 | 0.00736744 |
| SYS1-DBNDD2 | -2.879207869 | 0.006961189 |
| AC090673 | -2.666784131 | 0.006957799 |
| BSPRY | -2.943042032 | 0.006739302 |
| AC118553 | 3.384831758 | 0.006399731 |
| KCNH3 | -2.753097032 | 0.006070469 |
| ADGRE2 | -2.777557726 | 0.005906203 |
| AC005280 | 2.158488841 | 0.005845398 |
| GAP43 | -2.04033811 | 0.00565501 |
| AL353572 | -3.983601887 | 0.005389369 |
| KRT8P14 | -2.252043406 | 0.005301015 |
| NECTIN4 | -2.071981187 | 0.004986447 |
| AL139156 | -2.389139086 | 0.003947819 |
| AL359091 | -2.001980275 | 0.003831656 |
| TENT5B | -2.197998751 | 0.003766816 |
| AL359232 | -3.155996174 | 0.003647358 |
| RPSAP17 | -2.154842647 | 0.003284491 |
| LCE1F | -2.694510005 | 0.003066951 |
| CCDC17 | 2.127212537 | 0.002905455 |
| SH3TC2-DT | -4.031775064 | 0.002666029 |
| Z83844 | -4.955049037 | 0.002213318 |
| LINC01204 | -2.56411091 | 0.002112839 |
| PCAT1 | -2.566090346 | 0.001740541 |
| AC005865 | -2.212096689 | 0.001728277 |
| SLC9A3 | 4.181153523 | 0.001726205 |
| AL359546 | -2.198542187 | 0.001587506 |
| HKDC1 | -4.767715199 | 0.001218492 |
| FNDC11 | -2.690575922 | 0.001173517 |
| AC009163 | -7.019838839 | 0.001097199 |
| ARPIN-AP3S2 | 4.852118784 | 0.001068679 |
| RPSAP52 | -2.036560474 | 0.001035926 |
| PRODH | -3.057823997 | 0.000991185 |
| TRIM74 | 2.374537129 | 0.000966876 |
| AC138028 | 2.010632799 | 0.000938261 |
| CACNA2D1 | -2.203874132 | 0.000901476 |
| AC097518 | -2.049351827 | 0.000876275 |
| SNORA77B | -4.309811337 | 0.000845674 |
| WASH5P | -3.072797247 | 0.000843792 |
| MYOM1 | 2.041559475 | 0.000830724 |
| COL5A2 | 2.210846116 | 0.000793329 |
| CENPS-CORT | -3.017845922 | 0.000756605 |
| AC122697 | -4.740271714 | 0.000663165 |
| RN7SKP11 | 2.134476691 | 0.000651391 |
| HMGA2-AS1 | -2.165881212 | 0.000578554 |
| AL031118 | 2.674025399 | 0.000556428 |
| SEC31B | 2.014142026 | 0.000542679 |
| AC010327 | -6.427303095 | 0.00051574 |
| LCE1E | -4.110579818 | 0.000511758 |
| ADAP2 | 2.296480984 | 0.000390838 |
| AC097263 | -2.20584524 | 0.000365505 |
| RNF32-AS1 | 2.017846443 | 0.000322596 |
| AL645608 | 2.043794247 | 0.000322212 |
| CCDC74B | -2.004090203 | 0.000318703 |
| RCN3 | -2.404272199 | 0.000249599 |
| PIP5K1B | 2.255943495 | 0.000229417 |
| LCN15 | -6.511264345 | 0.000209759 |
| SYTL1 | -2.332651525 | 0.000208779 |
| CTSK | -3.481871741 | 0.000206838 |
| SARDH | -3.466940974 | 0.000198121 |
| KIF1A | -6.626080665 | 0.000187018 |
| AC007842 | 2.020896622 | 0.000186664 |
| TLR3 | -2.2922559 | 0.000185015 |
| DERL3 | -2.038787138 | 0.00017137 |
| FAM187A | -2.079699496 | 0.000171244 |
| AL138999 | -2.092587228 | 0.000147087 |
| IGIP | 2.110466898 | 0.000147048 |
| KCNH6 | 2.327463903 | 0.000144864 |
| ZNF672 | -3.605899028 | 0.00012878 |
| TUBB2B | 2.233718435 | 0.000125008 |
| NKAIN1 | -2.288559031 | 0.000124058 |
| AL139220 | 2.124480186 | 0.000123637 |
| USP2-AS1 | -2.848695217 | 0.00012138 |
| AC024580 | 2.119279575 | 0.000117191 |
| AL596244 | 2.429054562 | 0.000115093 |
| AL139260 | 2.22186202 | 0.000102871 |
| TNFSF15 | -2.314262472 | 9.63671E-05 |
| CTSV | -5.543216429 | 8.1651E-05 |
| HAPLN3 | 3.093064984 | 7.57106E-05 |
| LIPH | 2.011983019 | 7.28491E-05 |
| HSD3B7 | -2.143202203 | 6.89343E-05 |
| AC087501 | -2.093805838 | 6.82637E-05 |
| FAM222A-AS1 | -2.030010395 | 6.50105E-05 |
| RAD9B | -2.137903264 | 6.41702E-05 |
| MIR31HG | 2.009185113 | 5.76129E-05 |
| LINC01224 | -4.769397522 | 5.18244E-05 |
| CDRT4 | 2.307045993 | 4.55121E-05 |
| NAT16 | -2.097342907 | 4.07442E-05 |
| MYOSLID | -2.495575874 | 4.07228E-05 |
| AC027307 | -2.385698339 | 3.82281E-05 |
| TAFA3 | -2.010353718 | 3.64579E-05 |
| TMEM121 | -2.712031478 | 3.50369E-05 |
| TBX6 | -2.448807856 | 3.34524E-05 |
| AC007728 | 2.45956857 | 3.0631E-05 |
| AC104825 | 2.033868019 | 2.52775E-05 |
| CFAP70 | 2.287858326 | 2.42887E-05 |
| SPATA12 | -2.201369163 | 2.4264E-05 |
| ZNF664 | -2.235941082 | 2.21142E-05 |
| FCGBP | 2.651995807 | 2.18673E-05 |
| MFAP2 | 2.235261735 | 2.13015E-05 |
| FAM81A | -2.030643514 | 2.07886E-05 |
| SEMA3B | 2.19194221 | 1.89382E-05 |
| ZCWPW2 | 2.30454887 | 1.88761E-05 |
| IL11RA | 2.770885796 | 1.63814E-05 |
| POLE2 | -2.234142217 | 1.46164E-05 |
| ZNF467 | -2.220333227 | 1.30427E-05 |
| DCHS1 | 2.017873494 | 1.1148E-05 |
| FLG | -6.989382062 | 1.07835E-05 |
| CFAP53 | 2.400507187 | 9.75041E-06 |
| LINC01812 | -2.293264283 | 8.88326E-06 |
| DIXDC1 | -2.147839473 | 7.76316E-06 |
| OVGP1 | 2.119937768 | 7.62333E-06 |
| CDH15 | -2.208638409 | 7.61705E-06 |
| ZFPM2-AS1 | 2.583450533 | 7.29172E-06 |
| FHAD1 | -2.211651393 | 6.65761E-06 |
| OAS2 | -2.680742555 | 5.20228E-06 |
| PCLO | 2.472900851 | 4.61861E-06 |
| INHBE | -2.787702179 | 4.09515E-06 |
| CNTN4 | 3.094121039 | 4.07879E-06 |
| AC011462 | -7.126616945 | 3.71767E-06 |
| AC020661 | 2.207451676 | 3.58868E-06 |
| SLC2A4 | -2.724087552 | 3.48816E-06 |
| YJEFN3 | 2.308324258 | 2.30755E-06 |
| TRBV13 | -7.278255115 | 2.27633E-06 |
| SNORD17 | -2.726989234 | 2.0276E-06 |
| UNC13D | 3.256696539 | 1.29606E-06 |
| FAM217B | 2.758185134 | 1.25491E-06 |
| DNAAF3 | -2.046612352 | 1.24747E-06 |
| MRPS2 | -2.044293005 | 8.05745E-07 |
| AC241952 | 2.385193936 | 6.20886E-07 |
| AC087741 | 2.592489645 | 5.92689E-07 |
| AC068587 | -3.341234121 | 5.80439E-07 |
| SEMA3C | 2.576359004 | 5.47499E-07 |
| RAD54L | -2.15124983 | 5.4471E-07 |
| AC106845 | -2.908487255 | 5.1586E-07 |
| AC009108 | 2.30661888 | 4.77816E-07 |
| MIR22 | 2.523183021 | 4.62975E-07 |
| SUGCT | -5.511401153 | 4.30706E-07 |
| STARD8 | -3.278768994 | 3.96288E-07 |
| ANKRD24 | 2.303118281 | 3.92475E-07 |
| SIPA1L2 | 2.247772491 | 3.57636E-07 |
| DNAH10OS | 3.315308436 | 3.16887E-07 |
| AC046134 | 2.564275076 | 3.06507E-07 |
| LRRC45 | -2.063806851 | 2.86872E-07 |
| TFR2 | -2.138709739 | 2.82415E-07 |
| PARD6G-AS1 | -2.514586765 | 2.80447E-07 |
| UCKL1-AS1 | 2.922571961 | 2.70733E-07 |
| NRM | -2.127238346 | 2.65718E-07 |
| LGALS3BP | -2.091710113 | 2.62626E-07 |
| FGF7P6 | 2.131086771 | 2.09101E-07 |
| AL138689 | -3.853595197 | 1.99682E-07 |
| SPAG5-AS1 | 2.114962686 | 1.3969E-07 |
| MSN | -2.000599009 | 1.30113E-07 |
| SLC25A10 | -2.079837563 | 1.10793E-07 |
| AC092143 | 3.032594316 | 1.09364E-07 |
| ACTL8 | -7.683191273 | 9.60944E-08 |
| AKR7A3 | -2.103281125 | 8.92269E-08 |
| ANKFN1 | 3.087341061 | 7.59286E-08 |
| SNHG20 | -2.18742796 | 4.76305E-08 |
| CASC19 | -2.798914949 | 4.64899E-08 |
| LINC02086 | 2.162724702 | 4.60972E-08 |
| CTSF | -3.351849173 | 3.35467E-08 |
| TRPC1 | 2.285780347 | 3.13662E-08 |
| AC005586 | 2.561510907 | 3.10565E-08 |
| ARRDC4 | 2.007013623 | 2.82907E-08 |
| UPK3BL1 | -3.948983057 | 2.66849E-08 |
| ASIC3 | 2.478298357 | 2.5372E-08 |
| HELLS | -2.014000712 | 1.69506E-08 |
| LARGE2 | -2.262860094 | 1.63048E-08 |
| ELFN1-AS1 | -2.745736068 | 1.43149E-08 |
| ZBTB12 | -2.088261505 | 1.40108E-08 |
| ISYNA1 | 2.760274971 | 1.17857E-08 |
| IL17RE | -2.134086462 | 7.54247E-09 |
| EFR3B | 2.374473534 | 7.33225E-09 |
| PLCB2 | -2.288852479 | 7.06983E-09 |
| AC141586 | 2.047433835 | 6.97235E-09 |
| SERPINI1 | 2.541671876 | 6.54395E-09 |
| AC112777 | -2.154540565 | 6.05987E-09 |
| CALB2 | -2.732021778 | 5.54828E-09 |
| ACSM3 | -2.82569373 | 3.09654E-09 |
| PALM3 | -2.510283497 | 2.36481E-09 |
| H2BC15 | 2.409945219 | 2.16675E-09 |
| HGF | -2.357060361 | 1.46176E-09 |
| FER1L4 | 2.664225923 | 1.28441E-09 |
| MCM2 | -2.495856371 | 1.19572E-09 |
| OAS1 | -3.081954888 | 1.13683E-09 |
| LMCD1 | -2.127996648 | 1.01392E-09 |
| AC093525 | 2.073131226 | 8.68269E-10 |
| ZNF540 | 2.790949177 | 8.01358E-10 |
| AC007191 | -2.123705767 | 7.58314E-10 |
| NOP14-AS1 | 2.02157997 | 6.86781E-10 |
| LCN10 | -2.389473076 | 5.68537E-10 |
| MIR22HG | 2.338072506 | 5.06425E-10 |
| MX2 | -3.598239255 | 4.45132E-10 |
| FRY | 3.017384481 | 3.6587E-10 |
| AOC3 | 3.037802726 | 3.50583E-10 |
| HBE1 | -5.538007987 | 3.02228E-10 |
| NLGN3 | 2.921636581 | 2.83198E-10 |
| MYB | -3.580463932 | 2.5537E-10 |
| PDZRN3 | -2.122772993 | 2.28145E-10 |
| NTNG2 | -2.068923374 | 2.00159E-10 |
| CU633967 | -6.182664539 | 1.86002E-10 |
| RAB30 | 2.048332643 | 1.69055E-10 |
| UCP2 | -2.39247274 | 1.28806E-10 |
| CA12 | -2.180537708 | 9.85281E-11 |
| ZNF295-AS1 | -2.611596204 | 9.4747E-11 |
| AOC2 | 2.798801826 | 8.20454E-11 |
| SESN3 | 3.318639742 | 5.72235E-11 |
| KCND1 | 3.12425202 | 5.2066E-11 |
| RPLP0P2 | 2.400240132 | 4.85902E-11 |
| SORD2P | -2.038684443 | 4.54661E-11 |
| AC023593 | -2.812835638 | 4.17363E-11 |
| MT1L | -2.325141504 | 3.93946E-11 |
| H2BC5 | 2.162932997 | 3.72698E-11 |
| REL | 3.131550323 | 3.51053E-11 |
| CKMT1B | -2.315432591 | 3.44151E-11 |
| POLD1 | -2.092663521 | 3.16564E-11 |
| PMF1-BGLAP | -5.027928222 | 3.00815E-11 |
| ZSWIM5 | 2.017395534 | 2.81356E-11 |
| DDN | -2.712455456 | 2.48203E-11 |
| QRICH2 | 2.492305021 | 1.00723E-11 |
| AL139385 | 2.641201943 | 7.4508E-12 |
| MCM7 | -2.319360425 | 6.65491E-12 |
| TTN | -2.256712228 | 5.37127E-12 |
| AC026341 | -4.378374333 | 4.85077E-12 |
| HPDL | -2.840637711 | 3.03575E-12 |
| KIF9-AS1 | 2.78375845 | 2.91277E-12 |
| AC102953 | 2.36282791 | 2.83952E-12 |
| MCM5 | -2.133351218 | 2.74075E-12 |
| LRRN4 | -2.478073934 | 2.54055E-12 |
| SLC19A1 | -2.452297919 | 2.25313E-12 |
| AL162258 | -2.70721431 | 1.74796E-12 |
| PAICS | -2.078141352 | 1.71562E-12 |
| LIG1 | -2.061236508 | 1.65769E-12 |
| H2AC6 | 2.088562675 | 1.55221E-12 |
| SLC38A5 | -2.503369886 | 1.0996E-12 |
| H2BC8 | 2.841707531 | 8.11381E-13 |
| TRBV12-3 | -3.279494354 | 6.29436E-13 |
| IFITM1 | -2.3260823 | 6.10252E-13 |
| RHOB | 2.501782697 | 5.69994E-13 |
| SH3TC2 | -3.286535224 | 2.31783E-13 |
| GCNT4 | 2.482696292 | 1.23554E-13 |
| APLN | -3.036236402 | 1.0142E-13 |
| FGD3 | -2.733607821 | 9.53453E-14 |
| ATXN1 | 2.621399949 | 9.46642E-14 |
| TMEM217 | 3.612588344 | 8.97197E-14 |
| NR4A3 | 2.935615251 | 7.99934E-14 |
| BHLHA15 | -2.918895167 | 6.35321E-14 |
| ZNF425 | 2.067589701 | 2.55976E-14 |
| AC016876 | 2.893655547 | 2.05925E-14 |
| SEMA3D | -4.386602975 | 2.0377E-14 |
| C10orf55 | -3.91647225 | 1.6282E-14 |
| CLN6 | -2.118429865 | 1.20704E-14 |
| PARD3B | 2.033826651 | 1.14417E-14 |
| TP53INP1 | 2.15947121 | 9.94912E-15 |
| BNC2 | -3.237343913 | 3.2291E-15 |
| IPO4 | -2.570576853 | 1.42705E-15 |
| MYH16 | -5.407388056 | 1.10069E-15 |
| AL162253 | -3.548999155 | 1.02001E-15 |
| WDR4 | -2.650750681 | 6.08982E-16 |
| H2BC4 | 3.042201614 | 5.51181E-16 |
| TNXB | 4.980593166 | 2.80998E-16 |
| NES | -2.613991341 | 1.93876E-16 |
| MZF1-AS1 | 3.100027872 | 1.27417E-16 |
| KLF2 | 2.270297527 | 1.21956E-16 |
| CTSB | -7.090244874 | 1.16698E-16 |
| RGS4 | -2.846715155 | 1.07128E-16 |
| SMIM14 | 2.371461438 | 7.38227E-17 |
| PTGS2 | 2.311212289 | 1.42318E-17 |
| HAS2 | -3.959597165 | 9.63189E-18 |
| SDCBP2 | 2.61575836 | 2.91823E-18 |
| DOC2A | -4.399288515 | 1.81672E-18 |
| EGR2 | 2.499469925 | 1.46911E-18 |
| H4C8 | 2.596645104 | 7.19315E-19 |
| MCM3 | -2.35629598 | 5.62392E-19 |
| LINC00513 | 3.740716311 | 1.46669E-19 |
| GAL | -2.944323975 | 3.43698E-20 |
| LINC01358 | -2.738827934 | 1.04939E-20 |
| NT5E | 2.245820842 | 1.57139E-21 |
| ZNF778 | 3.124074923 | 9.31304E-30 |
| MAP1LC3C | -4.843532905 | 4.76588E-31 |
| CTSS | -3.636629404 | 2.11848E-33 |
| CTSO | -3.156096829 | 1.87306E-37 |
| SAMD11 | -2.295780234 | 6.73E-19 |
| NDUFC2-KCTD14 | -2.188021785 | 7.04E-19 |
| AC000093 | -2.111865556 | 5.44E-19 |
| YPEL2 | 2.724541829 | 6.44E-19 |
| AC087190 | 2.835898449 | 7.05E-19 |
| APOLD1 | 2.916851413 | 7.34E-19 |
